# Oscillating *Per*-Cision

**DOI:** 10.1371/journal.pbio.0060192

**Published:** 2008-07-29

**Authors:** Ying-Hui Fu

## Abstract

In a similar manner to humans, fly circadian clock proteins are regulated by multiple phosphorylation sites, which affect a protein's activity or stability. A suicide model for destruction of transcriptional machinery may explain a conserved mechanism that gives the circadian clock a chance to respond to varying cellular influences throughout the circadian day.

The field of behavioral genetics began nearly four decades ago, when Seymour Benzer's laboratory set out to identify circadian rhythm mutants in Drosophila melanogaster. The first of these was called *period*, and both short and long period alleles were found [1]. It was not until some years later that the mutant gene was identified and exploration of the molecular basis of the circadian clock began in earnest [2,3]. Over the years, forward screens in Drosophila have led to identification of a number of loci that contribute to circadian rhythm function with different phenotypes, including short and long periods and total arrhythmia [4]. Detailed investigations at a genetic and molecular level began to define the cellular and molecular basis of circadian rhythmicity. In its most basic form, the circadian clock of the fruit fly consists of transcriptional activators that turn on expression of two circadian and oscillating genes (*period* and *timeless*), which are translated into proteins (PER and TIM) targeted for degradation by phosphorylation. Physical interactions between PER and TIM regulate their movement to the nucleus, where they directly interact with the transcriptional activators and suppress the expression of their own genes [5]. These findings also established the repressor role for PER and TIM in the transcriptional feedback loop. The temporal lag from the transcription of these autorepressors—their translation, nuclear accumulation, and negative feedback until their degradation—requires around 24 hours (circadian), and therefore sets the speed of the clock. An interlocked positive feedback loop has also been characterized. It is remarkable that such a simple, yet elegant model could be the basis of regulation for something as critical as synchronization of behavioral and physiological rhythms to the dramatic changes in light/dark and temperature on planet Earth.

Forward and reverse genetic investigation in rodents revealed that most of the Drosophila clock genes have counterparts in mammalian systems, although some have multiple homologs [6]. Many studies in the last decade established a similar basic clock system for mammals [7]; comparison of the fly and mouse clock demonstrated many conserved features, but some differences have also emerged [8,9]. For example, the role of TIM appears to have been replaced by CRY in mammals.

In addition to the genes and proteins that were identified as constituting the core molecular clock in living cells, interest in the post-translational modification of core clock proteins grew, in part, because some of the core clock genes in Drosophila, mice, and humans are kinases and phosphatases that regulate the phosphorylation status of the circadian core clock proteins [10]. Cell biological studies in cultured cells and genetic experiments in Drosophila led to the idea that phosphorylation of PER protein targets it for degradation by the proteasome. This would have an effect on the time required to accumulate sufficient protein and on the maintenance of appropriate protein levels as a function of circadian time. Regulation of PER degradation thus represents a potential mechanism for setting the speed of the clock. This introduced a whole additional layer of regulation for this important biological process.

More recently, forward genetics became possible in the human circadian system and have led to identification of multiple mutations that yield circadian phenotypes in people [9,11,12]. One of these mutations was found in a human *period* homolog, h*PER2*, and the mutation altered the amino acid 662 from serine to glycine. This mutation is sufficient to shorten the internal period length for human carriers by one hour, and causes them to go to bed at early evening hours (5–8 P.M.) daily (familial advanced sleep phase syndrome or FASPS) [12]. In vitro studies of this mutation demonstrated that a phosphorylation cascade of four additional amino acids (S665, S668, S671, and S674) immediately downstream of the mutated residue by casein kinase I (CKI)δ/ε (mammalian double-time [DBT] homologs) leads to increased transcription of h*PER2*, indicating that this motif plays an important role for PER2 repressor function. This phosphorylation cascade of the region immediately C-terminal to serine 662 is dependent upon a separate priming phosphorylation event of serine 662. This makes the PER2 phosphorylation status in this region a two-stage event. The priming phosphorylation of S662 leads to the phosphorylation of the S665/668/671/674 motif, and when this happens, PER2 becomes a weaker repressor with increased stability (Figure 1A). When PER2 is hypophosphorylated due to the human mutation, it becomes a stronger repressor and destabilizes the protein (Figure 1B) [13,14]. The amino acid sequence around this region of hPER2 is highly conserved among mammalian PERs, but not in Drosophila PER.

**Figure 1:**
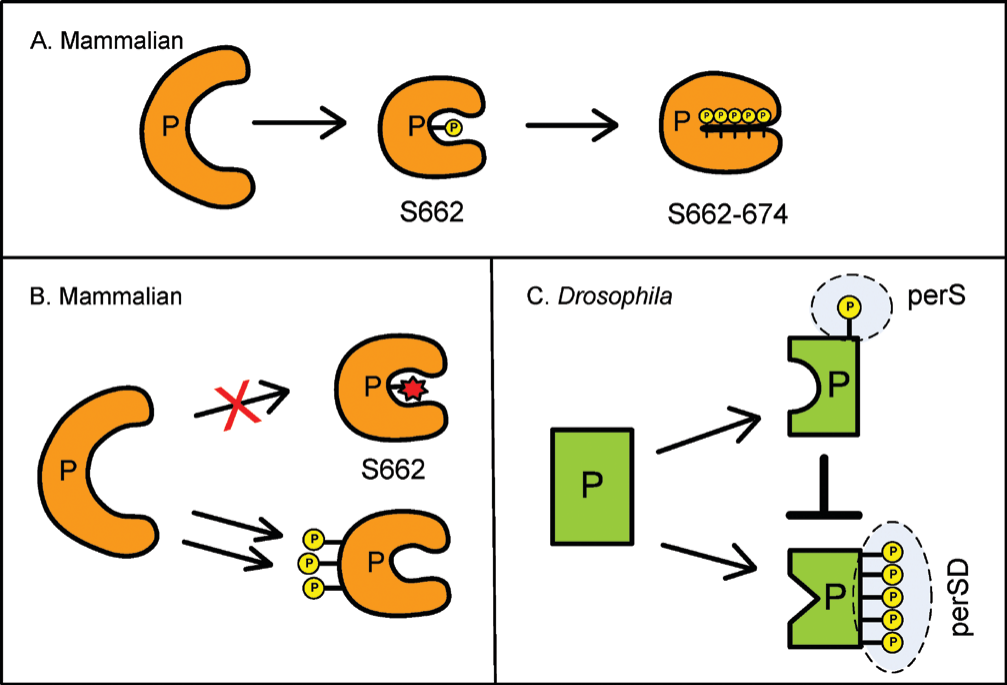
Phosphorylation Status of PER Regulates Its Repressor Activity (A) Mammalian PER2 is phosphorylated at serine 662, which then promotes the phosphorylation of S665/668/671/674. The completely phosphorylated S662–S674 PER2 is a weak repressor. (B) When mammalian PER2 serine 662 is not phosphorylated, it is a strong repressor, probably by facilitating modification (phosphorylation) on other PER2 motifs. (C) Drosophila PER can be phosphorylated at sites in either perS or perSD motifs. Phosphorylation in the perS domain has an inhibitory effect on the phosphorylation of perSD. Phosphorylation of perSD confers strong repressor activity.

The work of Kivimäe et al. reported in this issue of *PLoS Biology* characterizes a region in dPER that shares many features with the one described for mammalian PER2 S662/665/668/671/674 [15]. The authors report that phosphorylation status in both the N- and C-termini of dPER has no effect on its own repressor activity. However, two motifs in the middle of the protein, perS (per-short) and perSD (perS downstream), contain serine and threonine targets for DBT phosphorylation that do modulate the stability and repressor activity of PER. Kivimäe et al. propose that the phosphorylation of the perS domain acts to promote PER stability while reducing its activity as a transcriptional repressor (Figure 1C). Specifically, the phosphorylation state of perS (serine 589) can influence DBT activity on downstream targets within perSD that are required for PER function as a repressor. In this model, dephosphorylation of a serine in perS (S589) would promote DBT-directed phosphorylation of perSD, enhancing PER activity as a repressor and also destabilizing the protein. On the other hand, phosphorylation of perS (S589) depresses activity of DBT with respect to perSD, providing a more stable, but less active PER repressor. Although Drosophila perS/perSD and mammalian PER2 S662–S674 regions are not homologous, they are both found in a similar region of their respective protein. The layout of four phosphorylation sites (S604–S613) in the fly perSD resembles the CKIδ/ε phosphorylation motif found in human PER2 (S665–S674).

In the model proposed by Kivimäe et al., many features echo those described for the mammalian PER2 amino acids 662–674. Similar to mammalian PER2, perS and perSD of Drosophila PER are regulated by CKI phosphorylation. Phosphorylation of Drosophila perS and the hPER2 priming site (S662) both lead to more stable protein with lower suppression activity. However, some features seem to point in the reverse direction. Phosphorylation of mammalian PER2 S662 sets the stage for CKIδ/ε-directed phosphorylation of S665–S674, and hPER2 phosphorylated at serines 665/668/671/674 has a decreased repressor activity. In contrast, phosphorylation of Drosophila perS decreases DBT activity on perSD, and phosphorylated perSD has greater repressor activity. In addition, phosphorylation of S665/668/671/674 increases the stability of mammalian PER2. On the other hand, based on studies of mutant PER proteins in cultured cells and transgenic flies, phosphorylation of perSD is proposed to destabilize fly PER.

Despite the differences, both systems are reminiscent of the “suicide model” for transcription factors, where mechanisms for marking and destroying active transcription factors are integrated into the transcription activation process itself [16]. This coupling is achieved through coordinated action of the ubiquitylation and transcription machineries. One example of this is the yeast transcriptional activator GCN4, which is phosphorylated by the kinase Srb10, a component of the RNAPII complex [17]. This phosphorylation triggers its SCF (E3 ubiquitin ligase)-mediated ubiquitylation and subsequent proteolytic degradation. Degradation of the transcription factor follows soon after transcriptional activation. The recognition of the phosphorylated substrate by the SCF E3 ubiquitin ligase is mediated by F-box and WD40-containing proteins. In Drosophila, the F-box protein SLMB physically interacts with DBT-phosphorylated PER and promotes its proteasome-mediated degradation [18,19]. Flies with mutations in the *slmb* gene are arrhythmic. Cell culture studies have suggested that the mammalian ortholog of SLMB, ßTrCP, might play an equivalent role for mPER stability. Knocking down of ßTrCP or over-expression of a dominant-negative form of ßTrCP can efficiently stabilize PER proteins [20,21]. Interestingly, a mutation (*ovtm*) in an F-box protein FBXL3 was recently identified in mice that showed a long circadian period [22]. FBXL3 interacts specifically with the core clock repressor CRY and regulates its stability, suggesting a similar regulatory mechanism for mammalian PER and CRY.

The ability of the basal transcription machinery to mark an activator for destruction has led to the “black widow” or “suicide” model for activation, in which simply activating transcription is the signal for activator turnover. Here, for circadian transcription suppressors, the transcription machinery marks the repressors for destruction, where simply repressing transcription is the signal for its turnover (Figure 2). This model is particularly compelling for the circadian clock, since it can explain how multiple rounds of repression by a single repressor protein are prevented. This makes transcriptional regulation dependent on continuous reloading of transcription suppressors, affording flexibility in quickly responding to varying cellular influences throughout the circadian day. The fact that similar motifs, principles, and pathways are found (though in both similar and reverse directions) in different organisms suggests that a similar model for the regulation of transcriptional repressors is conserved between flies and mammals.

**Figure 2:**
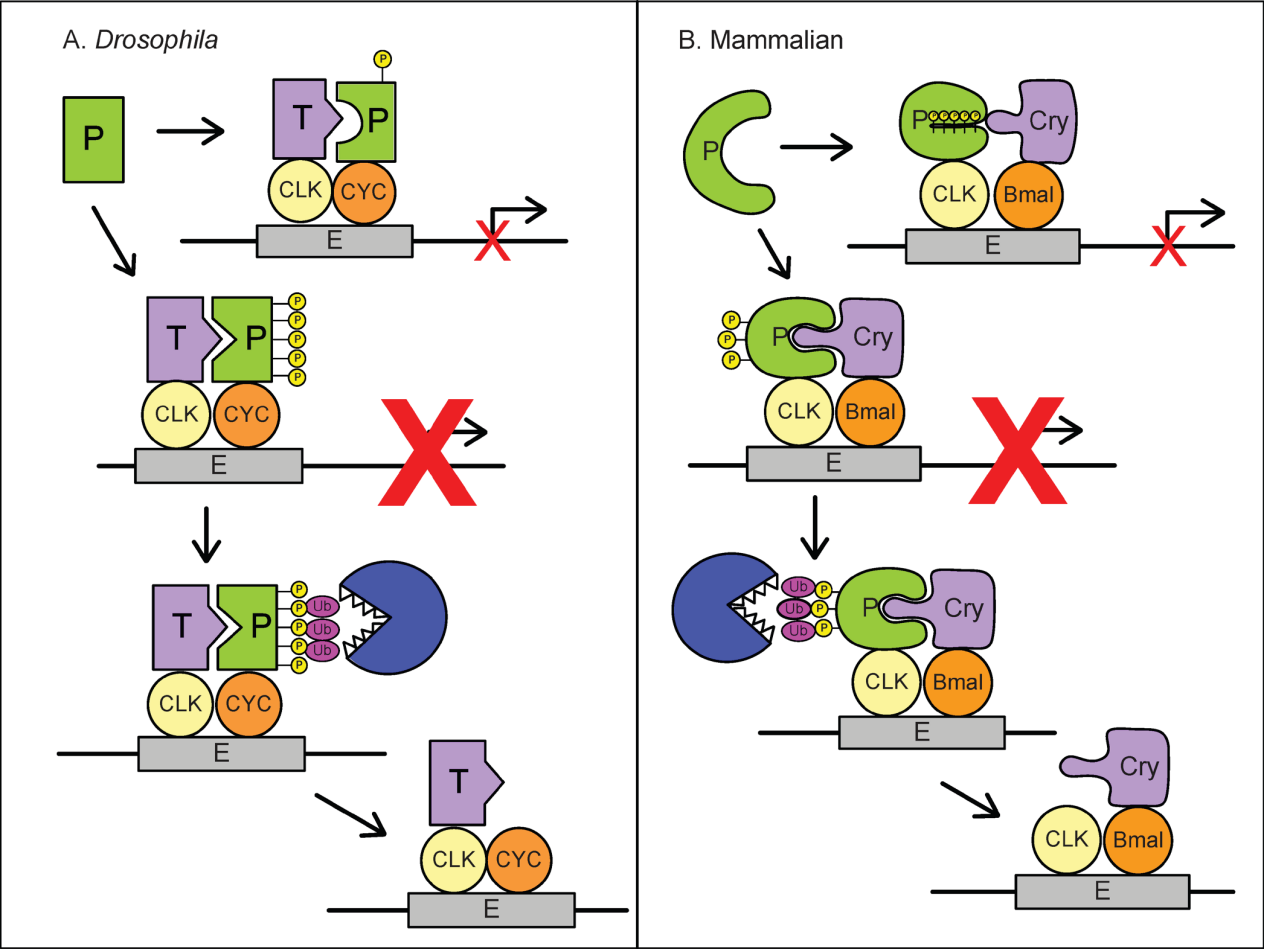
Suicide Model of PER Repressor (A) In Drosophila, phosphorylation of the perSD motif is associated with strong repression activity. When this strong repressor turns off the transcription by binding to the transcriptional machinery, it triggers proteasomal degradation of PER protein, thus facilitating its own turnover. (B) In mammals, PER2 is a weak repressor when S662/665/668/671/674 are phosphorylated. When unphosphorylated at S662–S674, PER2 is a strong repressor, and also becomes targeted for proteasomal degradation upon suppressing transcription.

As we approach 40 years since the dawn of the field of behavioral genetics, we have come a long way in understanding the intricate mechanisms of circadian regulation, with many conserved (but also different) mechanisms across species. The benefits of studying homologs in different systems are clearly demonstrated in these cases. The parallel multi-organismal studies of circadian biology have also offered an unprecedented example in revealing the fundamental nature of conservation through evolution for complex behavioral traits, and in revealing that basic mechanisms such as the “feedback loop” and “suicide model” have evolved both divergently and convergently for regulation of daily physiological and behavioral rhythms.

## Abbreviations

CKI: casein kinase I
DBT: double-time
PER: period
perS: per-short
perSD: per-short downstream
TIM: timeless
